# Regionally resolved cardiac metabolism using a dipole‐loop array coil for 7 T ^31^P‐MRSI

**DOI:** 10.1002/mrm.30492

**Published:** 2025-03-23

**Authors:** Jabrane Karkouri, Will Watson, Ria Forner, Jonathan R. Weir‐McCall, Tracy Horn, Marion Hill, Stephen Hoole, Dennis Klomp, Christopher T. Rodgers

**Affiliations:** ^1^ Wolfson Brain Imaging Centre University of Cambridge Cambridge UK; ^2^ Department of Cardiovascular Medicine University of Cambridge Cambridge UK; ^3^ UMC Utrecht Utrecht The Netherlands; ^4^ Department of Radiology University of Cambridge Cambridge UK; ^5^ School of Biomedical Engineering and Imaging Sciences Kings College London London UK; ^6^ Royal Papworth Hospital Cambridge UK

**Keywords:** ^31^P‐MRSI, array coils, heart energetics, heart failure, metabolic imaging, spectroscopic imaging

## Abstract

**Purpose:**

We introduce a novel commercial phosphorus‐31 (^31^P) dipole‐loop array coil, describing the coil hardware and testing its performance on phantoms. We used this coil to assess cardiac metabolism *per region* in healthy volunteers.

**Methods:**

B_1_^+^ field maps were simulated and compared to maps measured with a set of CSI sequences with varying voltages. Seventeen volunteers were scanned with 7 T phosphorus‐31 magnetic resonance spectroscopic imaging (^31^P‐MRSI). Reproducibility was assessed in nine of these volunteers. Strain was measured for six of these volunteers at 3 T.

**Results:**

Blood‐ and saturation‐corrected Phosphocreatine/γ‐adenosine triphosphate (PCr/ATP) ratios were measured for four regions of the left ventricle: 1.86 in septum, 2.25 in anterior wall, 1.41 in inferior wall, and 1.53 in lateral wall, respectively. These are in the expected range compared to previous studies. B_1_^+^ maps show good signal uniformity around the position of the heart (0.13 ± 0.06 μT/sqrt(W)). Intrasession and intersession coefficients of reproducibility were 0.22–0.88 and 0.29–0.79, respectively. Linear modeling shows that regional PCr/γATP correlates with circumferential strain but not radial strain. This requires corroboration by a larger study including patients with impaired function and energetics.

**Conclusion:**

Dipole‐loop array coils present a promising new approach for human cardiac ^31^P‐MRSI at 7 T. Their favorable B_1_^+^ uniformity at depth and specific absorption rate over loop arrays and improved SNR when combined with loops for reception could be beneficial for further clinical studies measuring energetics by ^31^P‐MRSI at 7 T. The new capability to assess PCr/γATP ratios across the whole left ventricle could enable clinical studies to investigate regional changes in cardiac energetics for the first time.

## 1 INTRODUCTION

Metabolic inflexibility is a key feature of the failing heart, and hence myocardial metabolism is a promising therapeutic target in heart failure.^1^, ^2^, ^3^ The ability to detect metabolic phenotypes and monitor treatment response in vivo is essential to the success of future translational research in metabolic targeting to treat heart failure.^4^

Phosphorus‐31 magnetic resonance spectroscopic imaging (^31^P‐MRSI) enables researchers to probe energy metabolism in vivo.^5^, ^6^, ^7^, ^8^, ^9^, ^10^, ^11^, ^12^, ^13^ Key compounds in energy metabolism such as phosphocreatine (PCr), adenosine triphosphate (ATP), and inorganic phosphate (Pi) can be quantified noninvasively by ^31^P‐MRSI. The PCr/γATP concentration ratio is a proven surrogate biomarker for effective cardiac energy metabolism.^6^, ^8^, ^9^, ^14^, ^15^, ^16^, ^17^, ^18^, ^19^, ^20^, ^21^

The cardiac PCr/γATP ratio has been shown to be a powerful predictor of mortality that is independent of New York Heart Association classification (a common clinical metric).^2^ Phosphorus metabolism changes in all forms of heart disease, and these changes occur early in disease.^4^, ^22^, ^23^ Phosphorus imaging is therefore a powerful and proven surrogate biomarker of heart failure that has been used in clinical research.^2^, ^14^, ^16^, ^20^, ^23^, ^24^, ^25^

To date, cardiac phosphorus imaging has not entered widespread use in clinical practice. What is lacking is sensitivity^26^ and spatial resolution to probe individual myocardial segments—that is, the coronary perfusion territories defined in the American Heart Association (AHA) 17‐segment model.^27^ Ultrahigh field MRI scanners can be used to increase sensitivity and spatial resolution of ^31^P‐MRSI.^28^, ^29^ In recent years, 7 T ^31^P‐MRSI has proven its value for applications in the heart and body.^14^, ^28^, ^29^, ^30^ Thus far, some of the biggest constraints at higher field strengths are due to the inhomogeneity of the B_1_^+^ field, which tends to drop so low in inferior cardiac segments that signals are undetectable. This is confounded by regulatory safety limits on specific absorption rate (SAR) and the need for a spectral bandwidth sufficient to cover all relevant ^31^P‐containing metabolites.

Surface coils cannot achieve the required flip angles to scan the inferior cardiac segments.^15^, ^28^, ^31^, ^32^, ^33^, ^34^, ^35^ Adiabatic pulses are not a viable alternative due to their high power deposition (SAR).^36^, ^37^ We previously reported a whole‐body birdcage design,^29^, ^38^, ^39^, ^40^ which performed well technically but requires invasive alterations to the MRI scanner that are not practical for larger scale or multisite applications.

In this study, we present a new dipole‐loop coil design^41^ combined with a loop receive array that enables 7 T ^31^P‐MRSI across the whole heart without needing any invasive changes to the MRI scanner. We describe the hardware setup, demonstrate performance in phantoms scans, and measure PCr/γATP ratios across the whole left ventricle in 17 healthy volunteers. We assess reproducibility in nine volunteers and compare to myocardial strain measured with 3 T MRI^42^, ^43^ in six volunteers.

## 2 METHODS

### 2.1 Hardware

All experiments were done on a Magnetom Terra scanner 7 T MRI (Siemens Healthcare, Erlangen, Germany) using a transmit (Tx)/receive (Rx) dipole‐loop array^44^ comprised of eight Tx/Rx hydrogen‐1 (^1^H) dipole elements, eight Tx/Rx ^31^P dipole elements, and 16 ^31^P Rx loops (Tesla Dynamic Coils, Zaltbommel, Netherlands). Due to limitations of the scanner software and maximum RF power output, we performed this study using only the central four ^31^P dipoles for Tx with all elements used for Rx, and with only one posterior ^1^H dipole for Tx with all elements used for Rx.

**FIGURE 1:**
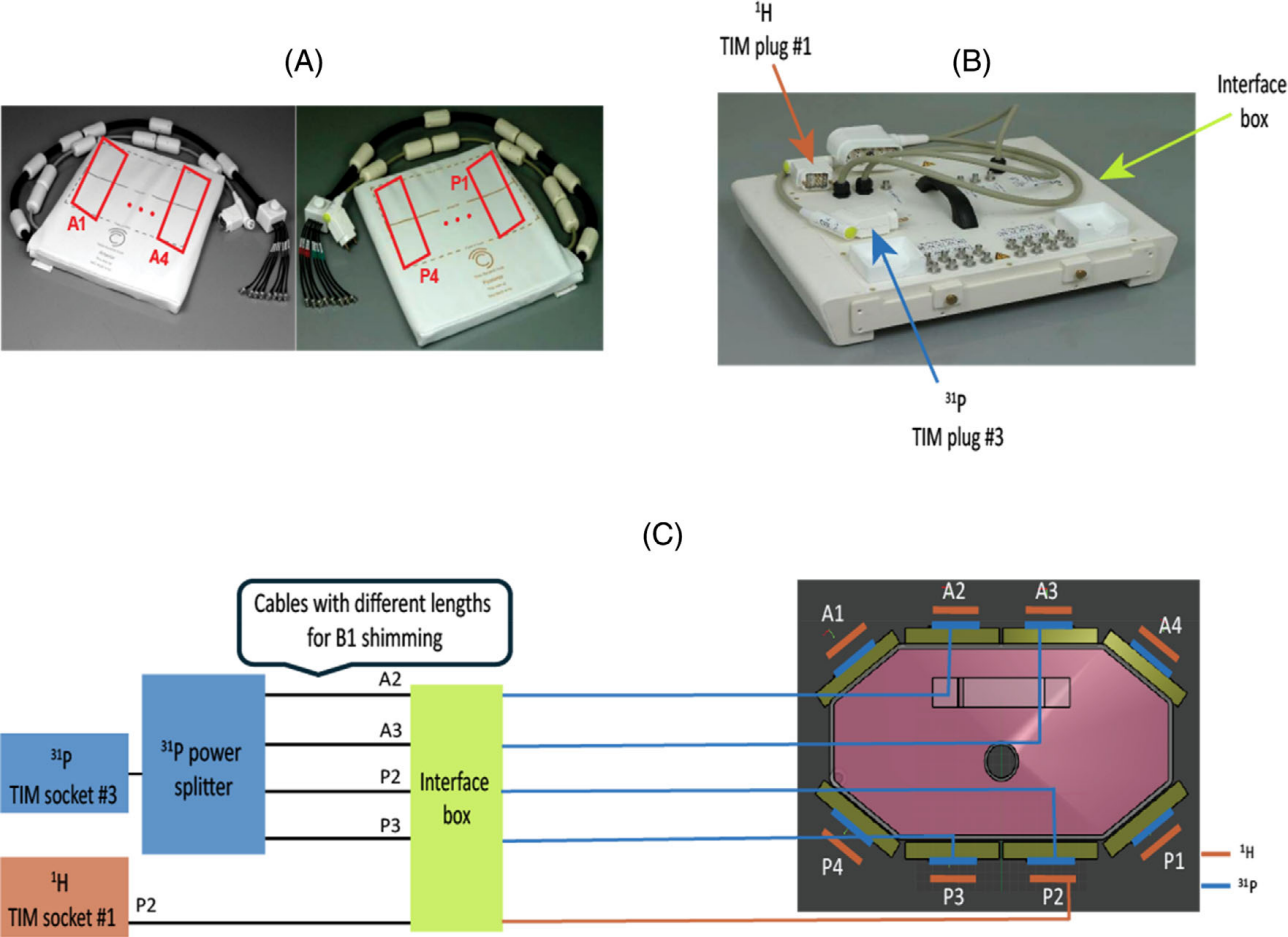
(A) Photograph of the coil showing the posterior and anterior elements. (B) Interface box connecting the coil to the scanner, which includes Tx/Rx switches and preamplifiers. (C) Position of the 8× ^1^H and 8× ^31^P Tx/Rx dipoles on the body phantom (right side). The ^1^H dipoles are stacked outside the ^31^P dipoles. Schematic of the coil interface electronics (left side). The scanner ^31^P output from TIM socket 3 is split four ways and fed to the central four dipoles for Tx (A2, A3, P2, and P3). All 8× ^31^P dipoles and 16× ^31^P loops are used for Rx. The scanner ^1^H output from TIM socket 1 is fed to 1× posterior ^1^H dipole for Tx; all 8× dipoles are used for Rx. This is due to limitations in the 7 T Terra software (Siemens Healthcare, Erlangen, Germany) that allow either parallel Tx or non‐proton scans in a single session. Cables with different lengths were connected from the dipoles (A2, A3, P2, and P3) to the back of the interface box for RF shimming. ^1^H, hydrogen‐1; ^31^P, phosphorus‐31; TIM, total imaging matrix; Tx/Rx, transmit/receive.

This dipole‐loop array coil consists of anterior and posterior elements as shown in Figure 1A. The dipole‐loop array coil is connected via Bayonet Neill‐Concelman cables to an interface box (Figure 1B) that contains the TR switches and preamps for each channel. The interface box is connected to the scanner total imaging matrix (TIM) sockets at the patient table. The interface has 2× TIM plugs (Figure 1C) for scanning in single Tx (sTx) mode with one ^1^H dipole transmitting, and a parallel Tx (pTx) TIM plug for scanning in parallel Tx mode with all eight ^1^H dipoles transmitting. Current “Terra” VE12U SP01 software only allows sTx scans in conjunction with ^31^P‐MRSI. When we receive the latest Terra. X software, this will enable pTx scans in conjunction with ^31^P‐MRSI using this coil.

Dipoles ^1^H and ^31^P are stacked on top of each other as shown in Figure 1C.

**FIGURE 2:**
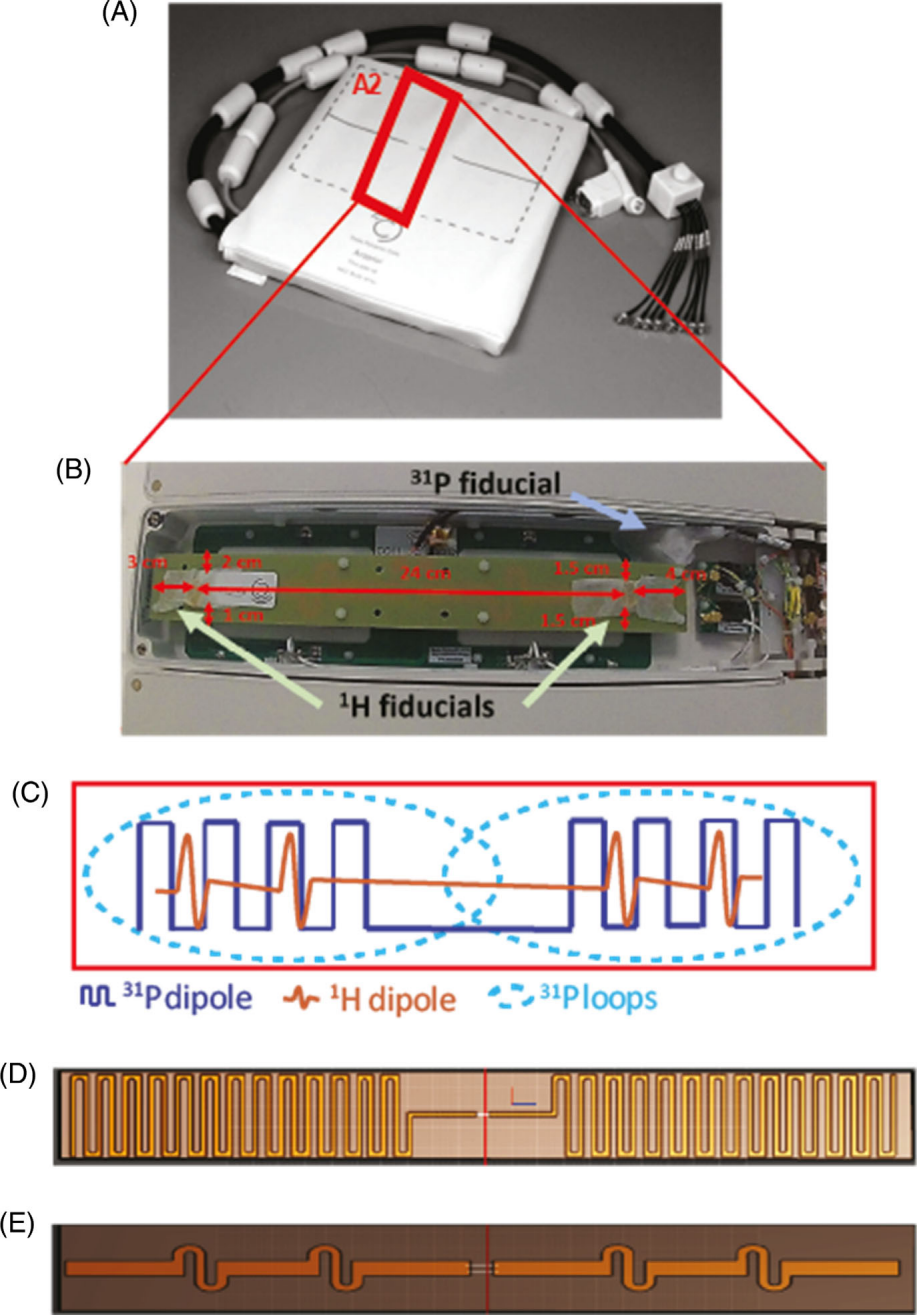
(A) Anterior element of the body array coil. (B) Zoom photograph of the A2 element of the coil. In this element, coil position marker (cod liver oil capsules, referred to as ^*1*^ *H fiducials*) were placed in the housing of the A2 element. A phosphorus calibration fiducial (see section 2.4.3 for content) was also installed. (C) Zoom illustration from within a coil element. Each housing from A1‐4 and P1‐4 contains one ^1^H dipole, one ^31^P dipole, and two ^31^P Rx loops. (D, E) The ^31^P and ^1^H PCBs are shown, respectively. PCB, printed circuit boards.

The coil comprises eight sealed element housings, each containing one Tx/Rx ^1^H dipole, one Tx/Rx ^31^P dipole, and two ^31^P Rx loops (Figure 2).

### 2.2 Electromagnetic simulations

**FIGURE 3:**
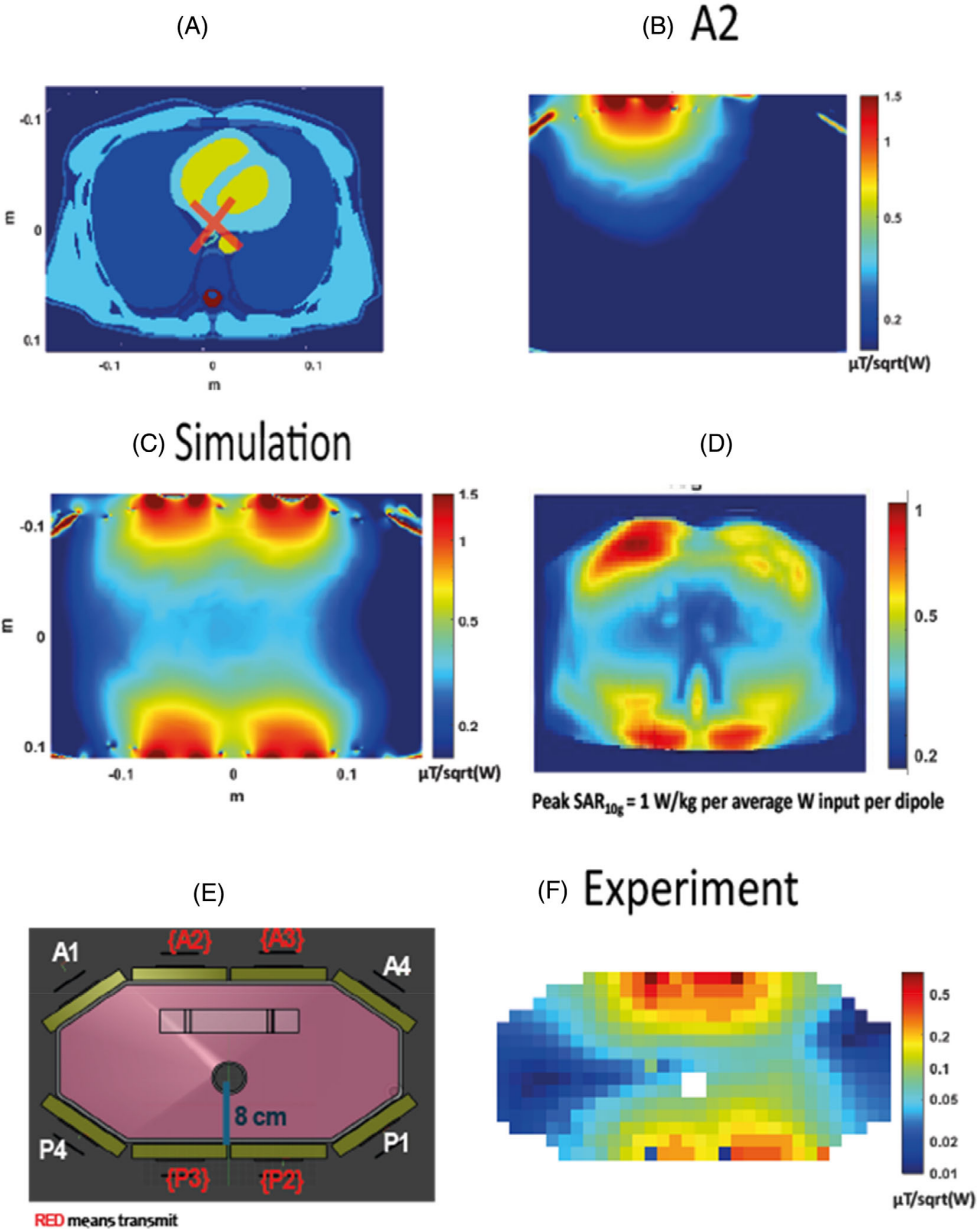
Comparison of the experimental B_1_^+^ map with the simulations. (A) A transverse slice of the Duke model (IT'IS Foundation, Zurich, Switzerland) used for simulations experiments. The RF shim target for the simulated B_1_^+^ map is outlined with a red cross. (B) ^31^P simulation results for transmitting only with the dipole A2. (C) The simulated map in μT/sqrt(W). (D) Peak SAR per average power input per dipole. (E) An illustration of the phantom used for the experiments. (F) The experimental B_1_^+^ map in μT/sqrt(W). SAR, specific absorption rate.

Electromagnetic field simulations of the dipole‐loop array coil were performed using Sim4Life (Sim4Life, Zurich MedTech, ZMT) to assess the safety and sensitivity of this coil design. Simulations were performed at 120.3 MHz on the Duke model (IT'IS Foundation, Zurich, Switzerland). The anterior and posterior elements of the coil were placed on the model, with the center of the antennas positioned over the center of the heart model (Figure 3A). Loops in the coil were tuned to 120.3 MHz and matched to 50 Ω. The B_1_^+^ and SAR over 10 g of tissue (SAR_10g_) were simulated 16 times each with 1 W power fed to a single dipole antenna (Figure 3D). From these simulations, the worst‐case SAR limits were determined by Tesla Dynamic Coils. We also combined simulation results for the four central ^31^P dipoles to model expected behavior in our experiments that used four ^31^P dipoles due to peak RF power limits on the 7 T Terra MRI scanner (Siemens Healthcare).

### 2.3 B_1_ ^+^ maps acquisitions

B_1_^+^ field maps were acquired on a phantom containing 26 L distilled water, 78 g sodium chloride, and 150 g dipotassium phosphate (ACROS Organics, Fisher Scientific, UK) (Figure 1C). The phantom has a central tube passing through it to insert additional small phantoms for testing.

Additionally, a 50 mL tube with 0.5 M phenylphosphonic acid (PPA) solution, which resonates at 17 ppm from the dipotassium phosphate frequency, was placed in the center of this phantom to enable for a single‐point B_1_^+^ value. The PPA solution tube was positioned such that it would align with the center of the antennas. Single‐point B_1_^+^ values were acquired for each dipole (A1‐4, P1‐4) with a series of FID scans using 5‐ms block pulses at voltages varying from 50 to 350 V in 25 V steps, 1 s TR, and the scanner reference frequency centered at PPA. Simulations were therefore compared with these single point B_1_^+^ values.

Although all 8× dipoles were tested for single‐point B_1_^+^ values, only 4× dipoles were used for the acquisitions of the B_1_^+^ maps and the in vivo applications.

A fixed hardware‐defined RF shim was determined for cardiac applications. To do so, the phantom shown in Figure 3E was used and rotated so that the hole in the center is closer to the anterior antennas (distance of 8 cm, which is about the distance from the chest wall to the center of the heart). The phase for the dipoles of interest (A2, A3, P2, and P3) for PPA signal from the central tube in the phantom were acquired with a 5 ms rectangular excitation pulse and 150V_rms_ peak voltage FIDs. Each FID was obtained with a single dipole directly connected to the scanner, with all other outputs of the power divider terminated to 50 Ω loads. The phase of each dipole was adjusted to match the others by swapping in different length cables (Figure 1C). This set of cables with optimized lengths was marked and secured and used for subsequent in vivo scans.

After RF shimming, B_1_^+^ maps were acquired transmitting on dipoles A2, A3, P2, and P3, and with a series of nine 3D CSI scans using a 32 × 32 × 8 matrix over a 45 × 45 × 30 cm^3^ FOV, a 4 ms block excitation pulse, a 1 s TR, and at voltages varying from 15 to 300 V.

Flip angles were computed by least squares fitting using lsqcurvefit (MatLab R2022b, MathWorks, Nattick, MA) to fit the intensities acquired at different voltages (per voxel for the CSI scans) to

(1) $$s=k\;\frac{\sin\alpha \times V_{\mathrm{RMS}}\;\left(1-e^{-\frac{\mathrm{TR}}{T1}}\right)}{1-\left(\cos\alpha \times V_{\mathrm{RMS}}\right)\;e^{-\mathrm{TR}/T1}},$$

where *s* is signal, *k* is a scaling factor representing coil Rx sensitivity and amount of substance and so forth, *α* is the flip angle per volt (°/V), TR is the repetition time (ms), T_1_ is the ^31^P longitudinal relaxation time (ms).^45^

Flip angle maps can be converted to B_1_^+^ field maps in Hz/V, μT/V, or μT/sqrt(W) by simple arithmetic based on the gyromagnetic ratio and the transmit pulse duration. A region of interest was generated with the heart model from Figure 3A to calculate average B_1_^+^ values at the depth of the heart, which were compared between simulations and experiments.

### 2.4 In vivo data acquisition

#### 2.4.1 MR scanning protocol

Seventeen healthy volunteers gave written consent and were recruited in accordance with ethical approval (nine males, eight females, age of 31 ± 8 ranging from 24 to 55 years, body mass index (BMI) of 23.9 ± 3.5 between 19.3 and 33.6 kg/m^2^).

All volunteers were scanned at 7 T with a scan time of approximately 1 h. Out of the 17 volunteers, six volunteers were also scanned at 3 T for approximately 30 min. Details about acquisition protocols are as shown in the following sections.

Nine volunteers out of the total 17 have taken part in a reproducibility study and were therefore scanned twice.

#### 2.4.2 7 T ^31^P acquisition protocol

The anterior and posterior parts of the coil were positioned on the body to align the center of the antenna with the center of the heart. The volunteers were scanned in a head‐first supine position.

**FIGURE 4:**
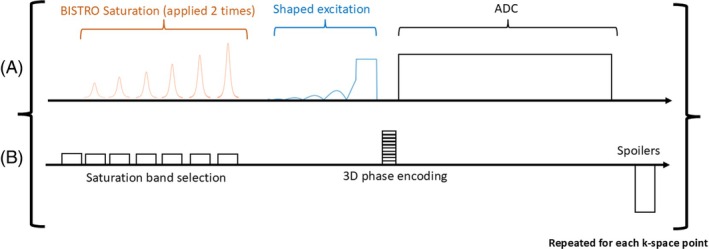
Chronogram of our custom MRSI sequence. (A) RF Tx/Rx (ADC) events. (B) Schematic gradient waveforms.

The 7 T protocol comprised anatomical localization using gradient echo scans (Siemens Healthcare cardiovascular “CV” pulse sequence), calibration scans, and ^31^P‐MRSI with our ultra short echo time CSI sequence^15^, ^34^ are shown in Figure 4. Localizers were acquired with 3.18 ms TE, 1600 ms cardiac TR (“true” TR between each excitation pulse of 5.9 ms), 350 × 350 mm^2^ FOV, an in‐plane resolution of 7.5 mm^2^, a slice thickness of 8 mm, and a 3.5° nominal flip angle (see Table S1).

To assess inter‐ and intrascan reproducibility, we performed a test–retest study. Session 1 followed the same 7 T protocol, plus a repeated 28 min CSI scan, leading to a total scan time of 1 h20 min. After a 5–10 min break, during which the subjects stood up and left the scanner room, the volunteers were scanned again. Volunteers were positioned afresh. Then, session 2 followed the 7 T protocol with a single 28 min CSI scan, leading to a total scan time of 50 min. Nine test–retest volunteers were scanned in reproducibility session 1, and six out of the nine volunteers were also scanned in reproducibility session 2 (see Figure S1).

#### 2.4.3 Data calibration and quality assessment

Our ^31^P fiducial contained phenylphosphonic acid (PPA) (ACROS Organics, Fisher Scientific, UK) dissolved in pure ethanol (Sigma‐Aldrich, St. Louis, MO) and with a pinch of chromium(III) acetylacetonate (Sigma‐Aldrich) to reduce T_1_, which was measured using nonlocalized inversion recovery scans and fitted to a three‐parameter model (T_1_ = 0.15 s). This was mounted inside the coil house after the B_1_^+^ field maps were completed (see Figure 2B).

For every in vivo scan, the 7 T acquisition protocol contains a set of calibration scans consisting of inversion‐recovery and voltage‐sweep FIDs sequences centered on the PPA frequency to find the T_1_ and the B_1_^+^ at the fiducial, as previously described.^28^ The inversion‐recovery FID acquisitions had inversion times incrementing from 25 to 1500 ms, a 5 s TR, and block pulses for excitation and inversion of 150 V each with a duration of 1 and 2 ms, respectively. The FID calibration sequences were acquired with increasing voltage from 50 to 250 V, a block pulse of 1 ms, and a TR of 2 s. The B_1_^+^ at the fiducial position was quantified using Equation (1) above.

#### 2.4.4 ^31^P MRSI measurements

The ^31^P MRSI pulse sequence is shown in Figure 4A,B. It consists of a selective saturation band module B_1_‐insensitive train to obliterate signal (BISTRO)^46^ comprised of six adiabatic full‐passage hyperbolic secant pulses. This is followed by a shaped excitation pulse,^33^ 3D phase encoding gradients, signal acquisition (ADC), and a spoiler gradient before the next TR. Noise measurements were made at the start of the whole acquisition before the first RF pulse for coil combination.

For the in vivo acquisitions, we used a 1 s TR, 450V_rms_ pulse amplitude to approach, depending on the cardiac segment; the Ernst flip angle based on the 3.05 s T_1_ of PCr^15^ (Ernst angle of 44° for a 1 s TR); and a 3D matrix size of 16 × 16 × 8 over a 30 × 35 × 30 cm^3^ FOV. The scan was 28 min long. B_0_ shimming was not performed for these acquisitions,^15^, ^28^ and we did not use respiratory or electrocardiogram gating.^47^ We used Hamming‐weighted *k*‐space filtering^48^ and acquired the ^31^P signal with a bandwidth of 5000 Hz and 1024 spectral points. We used two B_1_‐insensitive trains to obliterate signal saturation modules to reduce signal contaminations from the chest wall and the back muscles.

The placement of the CSI grid was done on the mid‐short axis localizer slice, rotating in‐plane and translating to give as many voxels as possible positioned at myocardium in the walls of the left ventricle.

#### 2.4.5 3 T MRI measurements

Six of the volunteers also had a 3 T CINE scan to assess cardiac function (3 T Prisma, Siemens Healthcare). The cardiac imaging protocol used the standard Siemens cardiac sequences consisting of Beat_TurboFlash_DB localizers performed in three orthogonal planes, followed by 2 chambers, 4 chambers, and short axis planes, and then BEAT_Cine_TruFISP cine sequences performed in 2ch, 3ch, 4ch, and short axis planes.^49^, ^50^, ^51^

### 2.5 Data analysis

Signals from multiple channels were combined with whitened singular value decomposition^52^ implemented online on the scanner.

Data were processed in MatLab R2022b (MathWorks) using an updated version of our Oxford spectroscopy analysis (OXSA) toolbox that supports Siemens 7 T Terra MRI scanners.^53^ Data were fitted with the advanced method for accurate, robust, and efficient spectral fitting. Our prior knowledge contained 11 Lorentzian peaks, including PCr, diphosphoglycerate, ATP peaks, inorganic phosphate, phosphodiesters, and phosphomonoesters. All of the scans were included in this analysis. All voxels from the ^31^P‐MRSI data were fitted. Four voxels from the ^31^P‐MRSI data were chosen in the equal anatomical locations for all subjects for further quantification analysis and comparison against 3 T functional measures from the mid‐interventricular slice. The voxels were positioned in the septal, anterior, inferior, and lateral wall segments. This was possible for every subject, whereas for subjects with smaller hearts it would not always have been possible to select six voxels—one for each AHA mid‐short axis segment.^27^ The SNR was computed following our usual approach (as implemented in the Oxford spectroscopy analysis toolbox^15^, ^53^) based on the Ernst et al. recommendations.^54^ The blood‐ and saturation‐corrected PCr/γATP ratio was computed using literature T_1_ values of 3.05 and 1.82 s for PCr and yATP, respectively,^15^, ^28^ and B_1_^+^ maps that were estimated by rescaling the phantom B_1_^+^ map using the per‐subject measured fiducial B_1_^+^ relative to the phantom‐measured fiducial B_1_^+^. This was used to compute the flip angle for each of the four voxels for each subject.

Cramér–Rao lower bounds were used to show the uncertainty in the metabolite concentrations. The coefficient of variation between volunteers for each regional PCr/γATP was calculated by dividing the standard deviation with the mean of the ratio.

Segmentation in the myocardium was performed following the AHA 17‐segment model.^27^ The AHA model contains six segments on the mid‐interventricular short‐axis slice. Segments 1 and 4 in this model correspond to the anterior and inferior regions of the basal myocardium. Segments 2 and 6 strain values were used for septal and lateral regions.

### 2.6 Statistical analysis

Short‐axis 3 T images were analyzed using Circle CVI42 (Circle Cardiovascular Imaging Inc., Calgary, Canada) to extract the circumferential strain (CS) and radial strain (RS). Note that *strain* is defined as a unitless percentage because it is defined as a relative change in length.^55^, ^56^

To investigate whether statistical analyses of PCr/ATP from all segments is viable in spite of strong differences in per‐segment mean PCr/ATP (see below), we applied stepwise linear modeling to test whether per‐segment PCr/ATP predicts circumferential strain (a measure of mechanical function^57^). Specifically, we applied the MatLab R2022b “stepwiselm.m” function (MathWorks) using the Akaike information criterion (AIC) to make an automated selection of the best model based on a combination of segment (a categorical variable), PCr/ATP ratio, and the nuisance parameters: BMI, age, and gender. We then repeated this for radial strain.

### 2.7 Assessment of reproducibility

Reproducibility of the protocol was assessed using three criteria:

- **Intersubject variability** was assessed by the mean and SD of the PCr/γATP ratio for all four cardiac wall segments, across all volunteers.
- **Intrasession variability** was assessed for volunteers that participated in reproducibility session 1 (nine volunteers) through the difference of the PCr/γATP for all four voxels between the first and second ^31^P‐MRSI scans.
- **Intersession variability** was assessed for volunteers that participated in both reproducibility scans 1 and 2 (six volunteers) through the difference of the PCr/γATP ratio for all four voxels between the first ^31^P‐MRSI acquisition of reproducibility session 1 and the ^31^P‐MRSI acquisition of reproducibility session 2.

Two coefficients of reproducibility (CR) were therefore computed. An intrasession reproducibility coefficient was computed as^28^:

(2) $${\mathrm{CR}}_{\text{intra}}={\mathrm{SD}}_{\text{intra}}\times 1.96.$$

An equivalent coefficient was computed from the intersession SD. Note that smaller values for the CR correspond to a more reproducible (better) method.

## 3 RESULTS

### 3.1 B_1_ ^+^ magnetic field simulations and experiments

**FIGURE 5:**
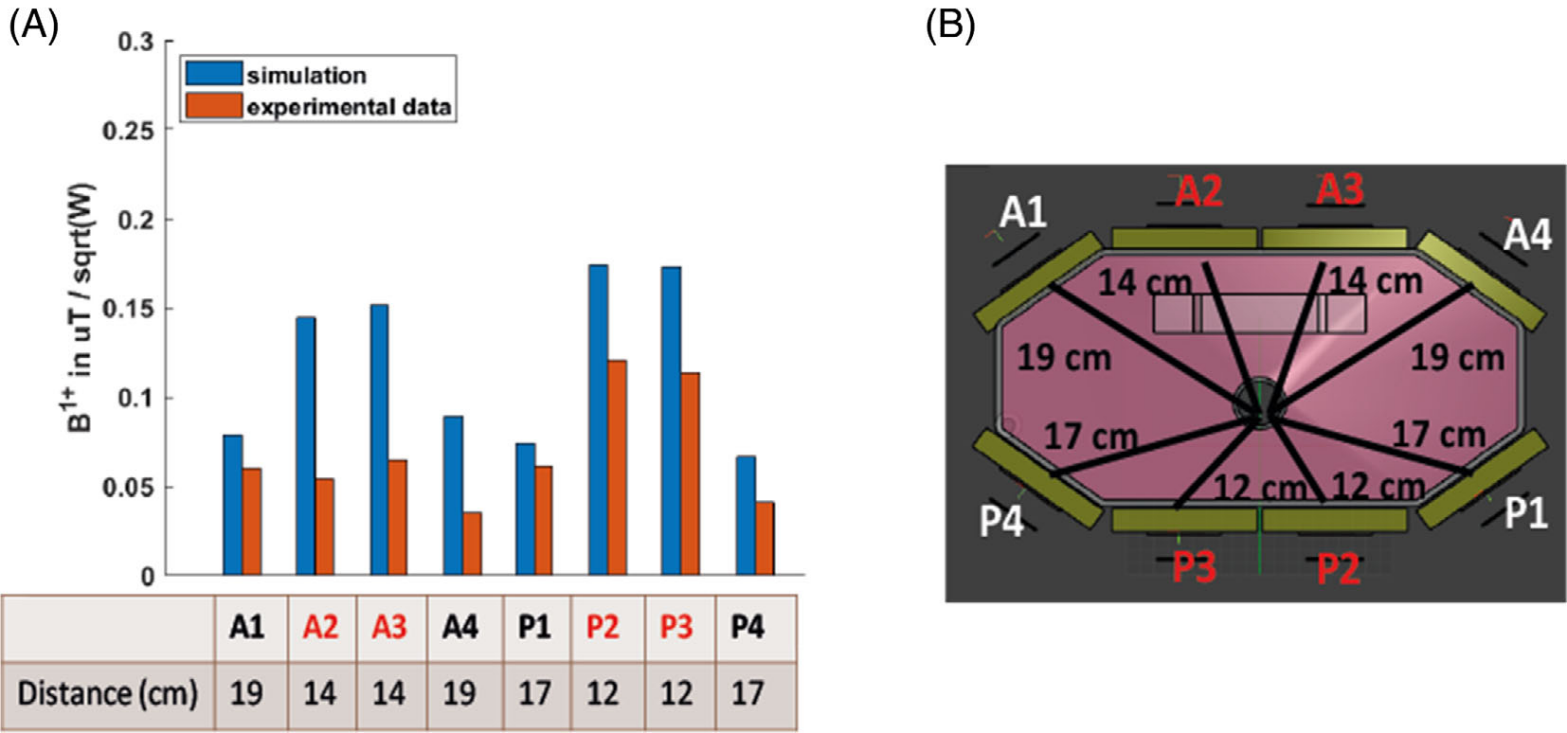
(A) B_1_^+^ value measured experimentally with a series of FID sequences of varying voltages and comparison with simulation results and (B) distances from the dipole to the center of the shaft. Only the dipoles highlighted in red were used for Tx in B_1_^+^ map and in vivo experiments.

Simulations have 1.4–2.2 times increased B_1_^+^ values compared to experiments (Figure 5), depending on the dipoles. This is expected because the simulations do not account for losses in the cables or interfacing electronics. Dipoles A2, A3, and A4 seem to be performing the worst compared to simulations, whereas dipoles P1 and A1 give closer B_1_^+^ values (Figure 5A). Distances from the dipoles to the PPA solution tube are shown in Figure 5B.

In Figure 3, the simulations are compared to experimental B_1_^+^ maps acquired from monopotassium phosphate in the main body of the phantom. Human voxel model simulations were performed for the Duke model, for which a mid‐transverse slice is shown in Figure 3A. The simulation of the B_1_^+^ Tx for the dipole A2 is shown in Figure 3B. Figure 3C shows a simulated B_1_^+^ map with a fixed RF shim and matched phases for all four dipoles to one voxel, as highlighted in Figure 3A. The combined map shows a decreasing sensitivity as we explore deeper into the body, and an average B_1_^+^ across the heart of 0.35 ± 0.10 μT/sqrt(W) (max 0.53 uT/sqrt(W), min 0.3 uT/sqrt(W)). The simulated SAR_10g_ is shown in Figure 3D. As expected, SAR_10g_ is highest closest to the antennas. The maximum safe power limit was 1.00 W/kg per average power input per dipole. This means that to comply with the third revision limits of IEC 60601‐2‐33 for local Tx coil SAR in first‐level mode, we must transmit no more than 20 W average power *per dipole* in any 6 min interval. The B_1_^+^ maps are shown in logarithmic scale. In Figure 3E, an illustration of the phantom is shown with the distance from the top of the phantom to the central hole marked. In Figure 3F, the experimental B_1_^+^ map is shown in μT/sqrt(W).

As in the simulations, the experimental B_1_^+^ maps show good uniformity around the position of the heart (Figure 3F), with an average B_1_^+^ at the depth of the heart of 0.13 ± 0.06 μT/sqrt(W), which is 64% less efficient compared to the simulations.

The shaped pulse used for in vivo acquisition was 2.3 ms long, corresponding to 0.5 ms effective block pulse duration^33^ at 450 V. This corresponds to 4.05 kW power input, and 8.27 μT on average given the B_1_^+^ experimental measurements. Converting from μT to actual flip angle using the pulse duration of 0.5 ms gives a flip angle of around 25.6° on average at the depth of the heart, which is lower on average than the Ernst angle of 44° computed for a 1 s TR and 3.05 s PCr T_1_.^15^

### 3.2 In vivo results

The results from the data calibration scans for each session are as follows; the B_1_^+^ values are 0.54 ± 0.053 μT/sqrt(W) and the T_1_ values are 0.148 ± 0.013 s on the fiducial. The SNRs of the spectra corresponding to a 90° at the fiducial position are 2428 ± 191.

**FIGURE 6:**
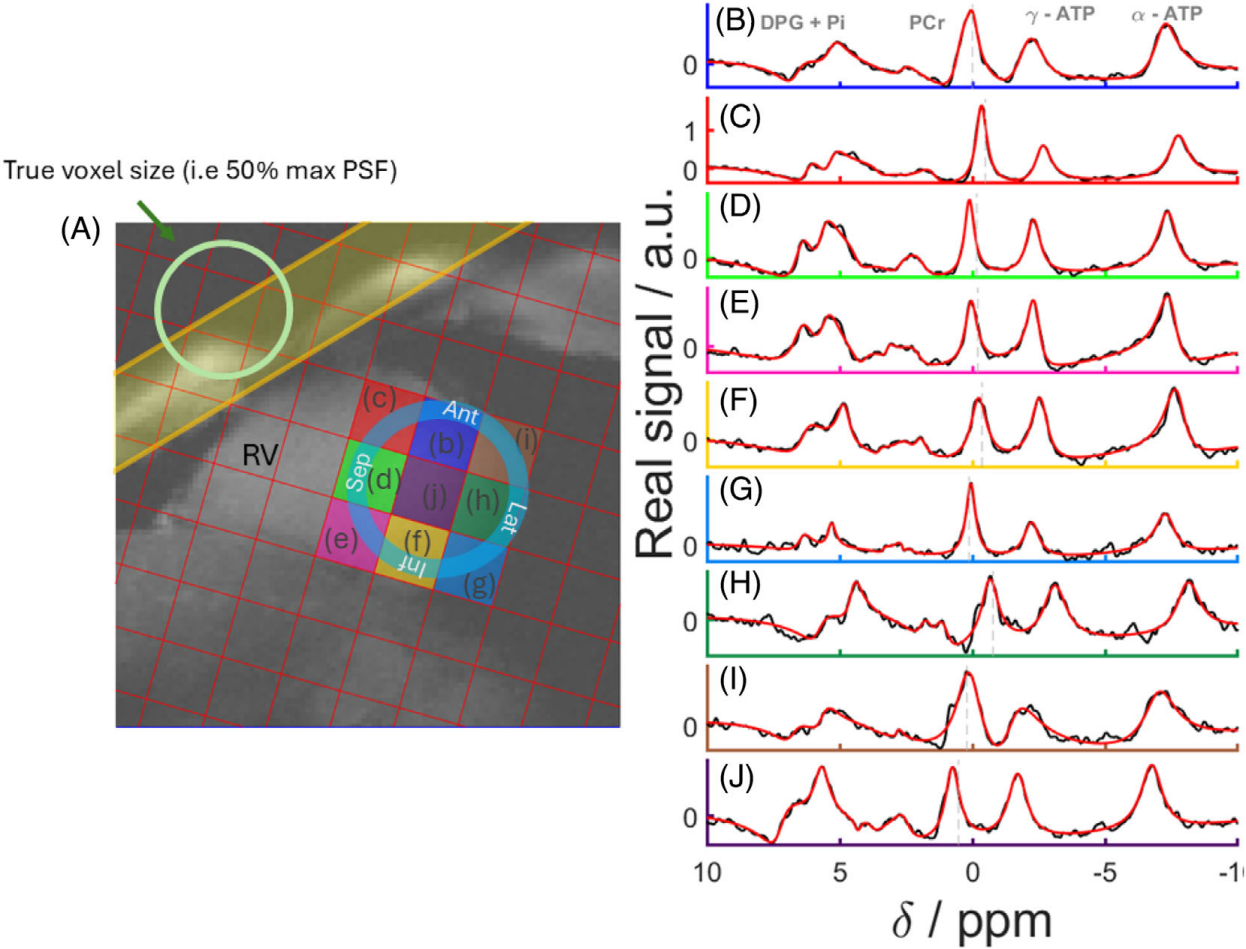
(A) Mid‐short axis GRE localizer acquired with breath hold with CSI matrix overlaid and PSF on the top‐left corner of the image. The BISTRO saturation band is shown in yellow. (B, J) Real part of spectra from the corresponding voxels (black) and corresponding AMARES fits (red). AMARES, advanced method for accurate, robust, and efficient spectral fitting; BISTRO, B_1_‐insensitive train to obliterate signal; GRE, gradient echo; PST, point‐spread‐function.

In vivo spectra across the myocardium for one participant are shown in Figure 6, which includes spectra from eight voxels covering lateral, inferior, septal, and anterior segments of the left ventricular myocardium. Spectra from the dipole‐loop array coil are of excellent quality (Figure 6, S2‐S5). Signals are clearly resolved for PCr, γATP, 2,3‐diphosphoglyerate, and phosphodiesters. Table 1 presents the PCr SNRs, linewidths, γ‐ATP SNR, PCr/γATP‐corrected ratio, and associated Cramér–Rao lower bounds per segments (anterior, septal, inferior, and lateral).

**TABLE 1:** Summary of PCr/γATP, PCr and ATP SNR, linewidth, the mean CRLB of the PCr/γATP ratio, and the CV of the PCr/γATP ratio between volunteers.

Region	Anterior	Septal	Inferior	Lateral
PCr/γATP	2.25 ± 0.66	1.86 ± 0.42	1.41 ± 0.20	1.53 ± 0.68
PCr SNR	218 ± 115	91 ± 60	61 ± 37	109 ± 84
PCr LW (Hz)	64 ± 30	39 ± 12	50 ± 26	91 ± 38
γATP SNR	95 ± 46	57 ± 38	46 ± 30	56 ± 37
CRLB	28%	11%	22%	33%
CV	0.35	0.21	0.14	0.51
CR_intra_	0.45	0.22	0.43	0.88
CR_inter_	0.79	0.45	0.29	0.65

*Note*: These values have been calculated for four different regions in the heart for all volunteers. For volunteers that participated in the reproducibility study, the first scan of session 1 was used to calculate the intersubject mean and SD in all voxels. The intra‐ and intersession coefficients of reproducibility are also reported, calculated with Equation (2).

Abbreviations: γATP, adenosine triphosphate; CR, coefficients of reproducibility; CRLB, Cramér–Rao lower bounds; CV, coefficient of variation; LW, linewidth.

In the septum, PCr SNRs were 91 ± 60 and the linewidths were 40 ± 12 Hz, whereas the γATP SNRs were 60 ± 38.

The blood‐ and saturation‐corrected PCr/γATP ratios were 1.86 ± 0.42 in the septum, 2.25 ± 0.66 in the anterior wall, 1.41 ± 0.20 in the inferior wall, and 1.53 ± 0.68 in the lateral wall. The optimum linear model for circumferential strain was (Figure S6):

CS˜1+PCr/ATP+Segment+Age+BMI

with *β* = −3.7 and *p* = 0.03 for PCr/ATP and *R* ^2^ = 0.727 overall.

An equivalent analysis for radial strain gave an optimum model (Figure S7):

RS˜1+Segment

(i.e., without PCr/ATP). The full output is provided in the Supplementary Information.

**FIGURE 7:**
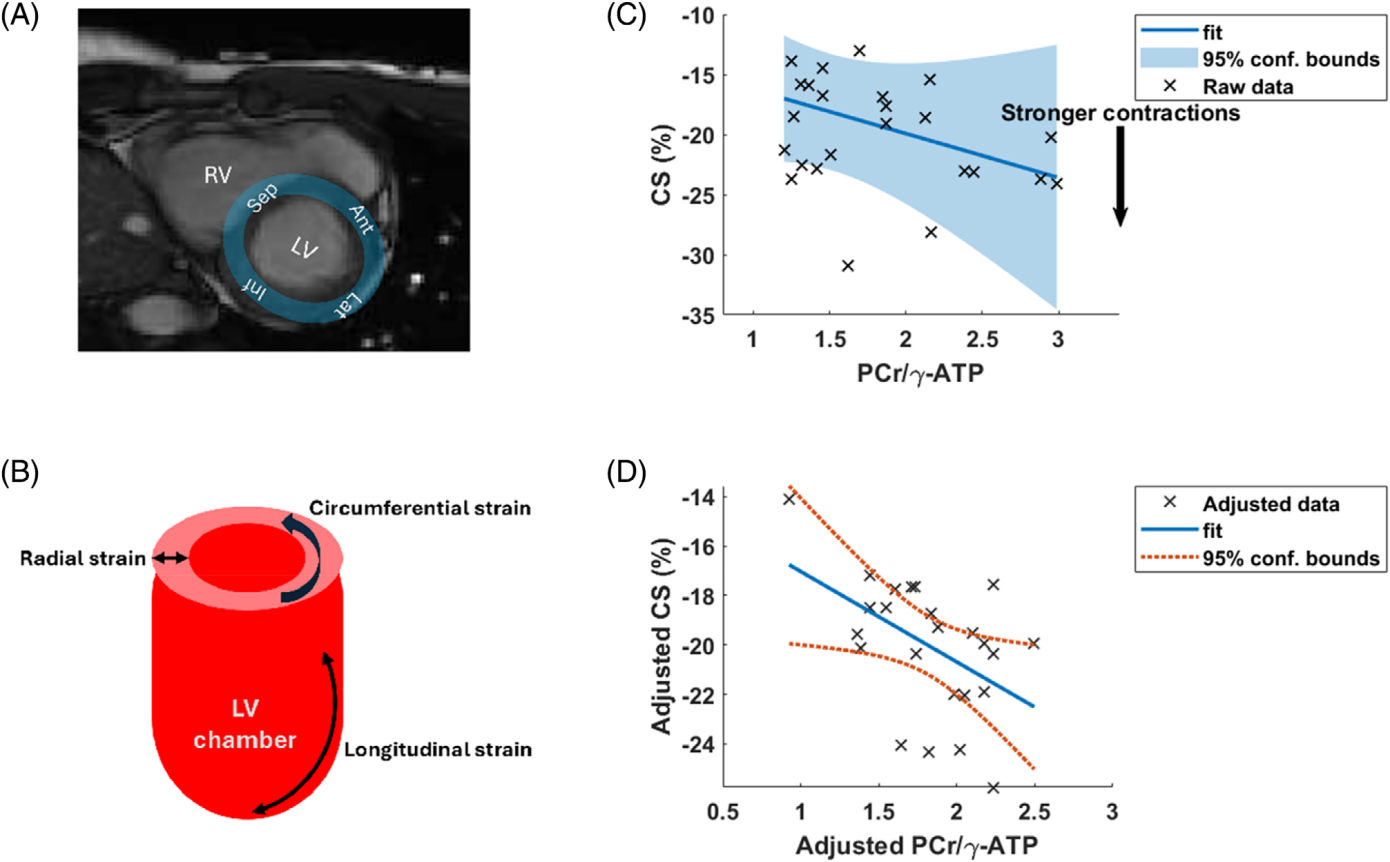
(A) 3 T short‐axis cine image from a volunteer showing the four cardiac segments. (B) Illustration of different types of strain measurement. (C, D) Results of stepwise linear modeling: (C) Plot of CS versus PCr/γATP) with all other parameters kept fixed, and (D) with the other parameters regressed out to show only the effects attributed specifically to CS and PCr/γATP. Plots (C, D) were made with the “plotSlice.m” and “plot.m” MatLab R2022b (MathWorks, Natick, MA) functions for linear modeling. γATP, adenosine triphosphate; CS, circumferential strain.

Results of the linear modeling are presented in Figure 7. In Figure 7C,D, plots of CS versus PCr/γATP are presented; in Figure 7C, all other parameters in the model kept fixed, and in Figure 7D, all other parameters regressed out to show only the effects attributed to CS versus PCr/γATP.

### 3.3 Assessment of reproducibility

**FIGURE 8:**
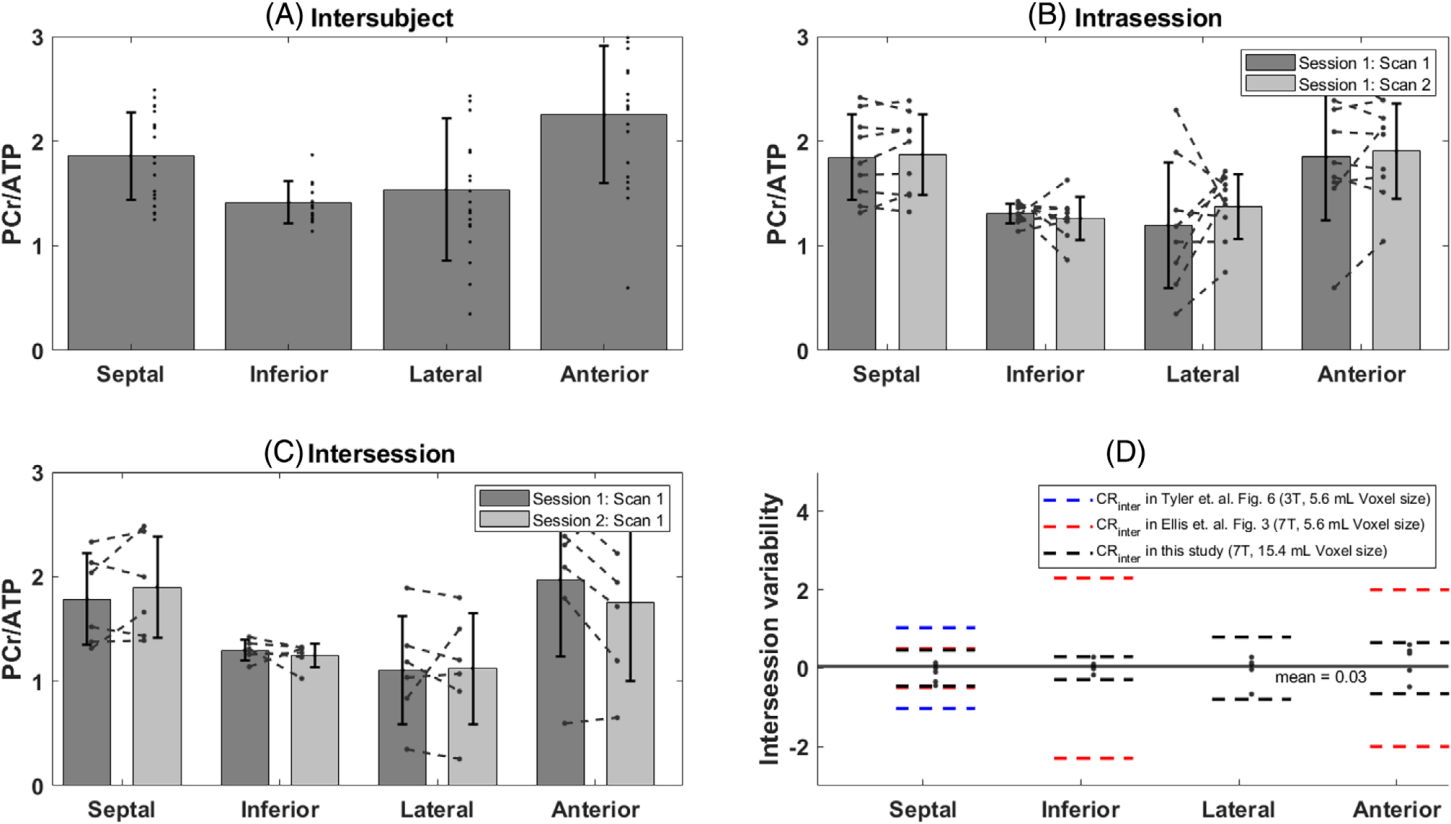
(A) Intersubject PCr/γATP bar plot in the septal, inferior, lateral, and anterior walls of the heart. For participants included in the reproducibility study, the PCr/γATP ratio from the first scan in session 1 has been used. (B) Intrasession difference of the PCr/γATP value between the first and second scan in session 1 for all subjects that participated in the reproducibility study and for each segment. (C) Intersession difference of the PCr/γATP value between the first scan of sessions 1 and 2. (D) Intersession variability defined by the difference of the PCr/γATP value between the first scan of sessions 1 and 2 and compared with coefficients of reproducibility from previous studies.^28^, ^33^

There were no significant changes in PCr/γATP between repeated measurement in the same subject in the four voxels of interest (Figure 8, Table 1). The intrasession CR was lowest for the septal and anterior voxels and highest for the lateral wall (Table 1, CR_intra_ of 0.22 and 0.43 in the septum and inferior wall versus 0.88 in the lateral wall). The intersession CR was lowest in the septum and inferior wall and highest in the lateral wall again (Table 1). Figure 8 illustrates the reproducibility efficiency of the method by showing the intersubject PCr/γATP in Figure 8A, the intrasession PCr/γATP difference in Figure 8B, and the intersession difference in Figure 8C. Figure 8D shows the intersession variability with a comparison of the CR with previous reproducibility studies in Ellis et al.^28^ taken from Figure 3 in their paper, and in Tyler et al.^33^ taken from Figure 6 in their paper.

## 4 DISCUSSION

### 4.1 Performance of the dipole‐loop array coil

We demonstrate the efficiency of a novel dipole‐loop array for cardiac ^31^P‐MRSI at 7 T. Transmit is achieved with Tx/Rx dipoles, and the signal is received with the eight dipoles plus an additional 16 Rx‐only loops.

Simulations and experimental measurements performed on the novel coil agree in terms of the B_1_^+^ coverage and uniformity. Simulations predict a higher B_1_^+^ compared to experiments, which is typical because the simulations do not account for cable losses and losses in the interface electronics. At the depth of the heart, the experimental B_1_^+^ maps show one‐third of the predicted field compared to simulations. This can be attributed to the loss due to cables (˜3 dB), partial mismatched antennas (˜1 dB), and electronic hardware (˜1 dB). On the experimental B_1_^+^ maps, a region of low B_1_^+^ is seen between the center and the left of the phantom. This could be due to a phase mismatch between dipoles in that area on the phantom because we adjusted the cable lengths based only on the single‐point measurements from the small PPA phantom in the central tube. For our application, we focused on the heart, where the B_1_^+^ was simulated as 0.13 ± 0.06 μT/sqrt(W) (mean ± SD).

This is comparable to the B_1_^+^ within the heart of 0.16 ± 0.075 μT/sqrt(W) simulated for our previous whole‐body insert ^31^P birdcage Tx coil and 16‐element Rx array prototype.^38^ The previous birdcage prototype would not fit into the Terra MRI scanner bore (Siemens Healthcare) because Terra added new rails to support the patient table, making this design unusable for our system. The dipole‐loop coil is more portable and may easily be inserted into a preexisting system setup without necessitating large hardware modifications because the coil interface simply plugs into the TIM sockets on the patient table of the Terra scanner. Although birdcage coils offer a more uniform B_1_ field, the ability to RF shim for differing target anatomies is limited, whereas a dipole‐loop coil can be optimally RF shimmed for the chosen target anatomy. The integration of Rx elements in the same housing as the Tx antennas ensures that the two are optimally geometrically decoupled and there is no variation with coil placement.

The T_1_ and B_1_^+^ values extracted during the in vivo acquisitions on the ^31^P fiducial have a low intersession variation (T_1_ of 0.148 ± 0.013 s, B_1_^+^ of 0.54 ± 0.05 μT/sqrt(W), and spectra SNR at 90° of 2428 ± 191). This suggests that the coil's Tx efficiency, phase, and loading are stable in between different scan sessions (8.7% variability for the T_1_, 9.3% for the B_1_^+^, and 7.9% for the SNR). This also suggests that coil loading is not that important for dipole designs because their bandwidth is relatively wide. Bench tests show that mismatch causes power reflection that will slightly reduce overall Tx efficiency but that it has little effect on the S_21_ phase and therefore does not significantly alter the required cable lengths for phase‐only RF shimming.

Recent emerging studies have shown interest in using dipoles for non‐proton applications^58^, ^59^; however, to the best of our knowledge we present here the first in vivo cardiac results using ^31^P Tx/Rx dipoles.

Figure 6 shows that the spectrum in the blood pool (Figure 6J) contains cross‐contamination from neighboring voxels, as highlighted by the PSF illustration of the CSI sequence used in Figure 6A.

The method presented in this study with this novel design has been shown to be reproducible on nine of the volunteers, with an intra‐ and intersession CR lower than 1 for all four voxels of interest. The intra‐ and intersession variability and the CR were the lowest in the septum and inferior segments and highest in the lateral and anterior walls (Figure 8). This goes in the line with the higher SD of the PCr SNR as shown in Table 1. Previous studies at 3 and 7 T have also shown that B_0_ inhomogeneities seem to be worse in the lateral and anterior segments of the heart, and that these areas are where the B_0_ field varies most across the cardiac cycle.^60^, ^61^, ^62^ The intersession CR is lowest for this study compared to previous studies by Tyler et al.^33^ at 3 T and Ellis et al.^28^ at 7 T as shown in Figure 8. It is important to note, however, that these studies have been performed with a surface coil and a higher spatial resolution, and therefore a direct reproducibility comparison is not completely possible.

### 4.2 Regionally resolved cardiac 7 T ^31^P‐MRSI

The in vivo ^31^P spectra from our 17 volunteers are of good quality, as shown for a selected volunteer in Figure 6. Despite a limitation from our current VE12U SP01 software on our 7 T Terra MRI scanner (Siemens Healthcare) only allowing single‐channel ^1^H Tx in conjunction with ^31^P‐MRSI, the stacked ^1^H dipole array coil gives improved coverage compared to our previous approach using a 10 cm loop coil for ^1^H‐MRI.^28^

The blood‐ and saturation‐corrected PCr/γATP ratio in the mid‐interventricular septum is consistent and lies in the expected range.^15^, ^26^, ^28^, ^33^, ^35^, ^63^ We are able to obtain blood‐ and saturation‐corrected PCr/γATP‐corrected ratios in the lateral wall for the first time.

Table 2 compares PCr/γATP values from this study against reports in the literature for different segments of the heart and at different field strengths. Our study is the first to use a dipole‐loop array coil design. This novel setup enables a better coverage of the body for regionally resolved cardiac ^31^P measurements.

**TABLE 2:** Comparison of PCr/γATP values between this study and the literature. Note that we are able to measure PCr/γATP in the lateral region of the heart.

References	Field strength	RF Coil	Cohort size	Acquisition sequence	Nominal voxel size	PCr/γATP septal	PCr/γATP inferior	PCr/γATP anterior	PCr/γATP lateral
Lamb et al.	1.5 T	Surface loop	16	3D ISIS	294 mL	1.4	–	–	–
Schaefer et al.	1.5 T	Surface loop	7	1D CSI	–	1.9	–	–	–
Pohmann et al.	2 T	Surface loop Tx + quadrature Rx	11	3D CSI	25 mL	2.05 mean, 0.31 SD, 1.25–2.56 range^a^			
Bakermans et al.	3 T	Surface loop	20	3D ISIS	512 mL	1.5–2.8	–	–	–
Tyler et al.	3 T	Surface loops	8	3D CSI	5.6 mL	2.1	1.8	2.4	–
Rodgers et al.	7 T	Surface loops	9	3D CSI	29.7 mL	1.7/2.1	–	–	–
Ellis et al.	7 T	Surface loops	10	3D CSI	5.6 mL 9.4 mL	1.70	3.0	2.7	–
This study	7 T	Dipole‐loop array	17	3D CSI	15.4 mL	1.86	1.41	2.25	1.53

^a^ Note that the Pohmann et al. study does not report per‐segment PCr/ATP but instead reports all voxels from all subjects pooled. The range was extracted from their Figure 5 using plotdigitizer.com. ISIS, image‐selected in vivo spectroscopy.

We suspect that the apparent strong variation in PCr/γATP values between segments is due to technical factors such as contamination of “anterior” spectra by adjacent skeletal muscle (due to the voxel point‐spread‐function and motion) and variations in B_1_^+^ across the heart.

As an exploratory analysis, we applied stepwise linear modeling to all six subjects with strain data and for all segments together. We included “segment” as a categorical variable to account for the strong differences in mean PCr/ATP between segments. For circumferential strain, the Akaike information criterion showed that PCr/γATP should be included in the optimum linear model (albeit with modest *p* = 0.03) to explain observed variation in circumferential strain, along with “segment,” age, and BMI. The same stepwise linear modeling approach only identified “segment” as explaining observed variation for radial strain.

The correlation of PCr/ATP, age, and BMI with circumferential strain but not with radial strain may be expected because previous studies have shown that circumferential strain is a more reliable biomarker^57^, ^64^; for example, a study of doxorubicin‐induced cardiotoxicity showed significant changes in circumferential strain but not radial strain.^65^

Meanwhile, a *t*‐test of circumferential strain versus PCr/γATP only in the septum was not significant (*p* = 0.15). This illustrates that regionally resolved ^31^P‐MRS could increase the statistical power of cardiac energetic studies even with strong regional variation in baseline PCr/γATP.

However, we caution that our study has few subjects and that the correlation to PCr/γATP is modest. Hence, we conclude that comparisons of regional strain and metabolism are presently inconclusive, requiring further patient studies.

The heart is known to be affected heterogeneously in many cardiomyopathies, with regional patterns of scarring and wall motion abnormality. To date, the ability to study the regional distribution of disordered metabolism, as well as the interaction of this with myocardial scarring and function, have been limited due to an inability to assess MRS at such a granular level. Enhanced heart coverage for ^31^P applications at 7 T will open the door to research studying the heart's energetic metabolism regionally. Our work complements recent measurements of regional cardiac metabolism by hyperpolarized ^13^C‐MRSI in volunteers,^66^ small animals,^67^ and in patients with heart failure.^68^ Cardiac ^31^P‐MRSI is a cheaper and logistically easier experiment, so it will be exciting to see whether these regional changes in patients with heart failure can be detected by cardiac ^31^P‐MRSI as well.

### 4.3 Limitations

Dipoles are known to generate a Poynting vector that is directed toward the body.^41^ At higher frequencies (i.e., at 7 T) and larger object sizes (body), this effect is enhanced.

Although the coil we present contains 8× ^1^H transceiver dipoles that are capable of excellent image quality using parallel transmit,^69^, ^70^ due to limitations in the VE12U SP01 software on our 7 T Terra scanner (Siemens Healthcare), we could only use 1× dipole in these scans. Consequently, our anatomical ^1^H imaging was suboptimal, requiring a separate short 3 T MRI scan to assess cardiac function and volumes. The newer Terra. X scanner model (Siemens Healthcare) removes this limitation but is not yet available in Cambridge.

Additionally, whereas the coil contains 8× ^31^P Tx dipoles, we chose to use only the central 4× dipoles in this study because of the limited 8 kW power available on 7 T Terra scanner (Siemens Healthcare). In the longer term, we hope to drive all 8× dipoles with a 35 kW amplifier, but that is beyond the scope of this study.

Additionally, the SNR for spectra from the lateral segment was low. This could have been due to low flip angles there, poor Rx sensitivity, motion‐induced signal dephasing—or to a combination of all of these effects.

Even though the novel setup described in this study offers a better coverage of ^31^P‐MRSI, this coverage comes with a limitation in terms of the voxel size that can be achieved within the same acquisition time as previous studies.^28^, ^33^ Indeed, a bigger CSI matrix size is needed in order to avoid contamination from the back and abdominal muscles.

Finally, the PCr/γATP ratio has been corrected for blood and saturation based on the flip angle reached on each segment (anterior, septal, inferior, and lateral), calculated with the experimental phantom B_1_^+^ map. However, there will be an additional error to that PCr/γATP ratio due to breathing and motion that has not been taken into account here. The B_1_^+^ field map may also vary per subject, which would also change the corrected PCr/γATP ratio. Based on our coil calibration scans on the ^31^P fiducial in vivo and the small B_1_^+^ variation in the fiducial, we do not expect the B_1_^+^ map to vary significantly.

## 5 CONCLUSION

Dipole‐loop array coils present a promising new approach for human cardiac ^31^P‐MRSI at 7 T. We evaluated the efficiency of such a setup with B_1_ simulations and experimental measurements. We confirmed technical stability between scans by monitoring signals from a ^31^P fiducial inside the coil housing. We report for the first time blood‐ and saturation‐corrected PCr/γATP concentration ratios in the heart's lateral wall. The reproducibility of the protocol and the setup has been evaluated on nine out of the 17 total volunteers, and we show a low intra‐ and intersession variability. This new capability could assist trials of therapies for heart failure that target cardiac energy metabolism.

## CONFLICT OF INTEREST

Christopher Rodgers receives research support from Siemens for another project.

## Supporting information


**Figure S1.** Diagram illustrating the different scans that volunteers have undertaken. Seventeen volunteers were scanned in total. Eight volunteers were scanned for the main study only and 9 were scanned for the main study and the reproducibility study.
**Figure S2.** ^31^P‐MRSI results for a volunteer with a body mass index (BMI) of 34 kg m^−2^. (A) Mid‐short axis GRE localiser acquired with breath hold with chemical shift imaging (CSI) matrix overlaid. (B–I) Spectra from the corresponding voxels. Both fit (in red) and raw data (in black) are shown.
**Figure S3.** ^31^P‐MRSI results for a volunteer with a body mass index (BMI) of 26 kg m^−2^. (A) Mid‐short axis GRE localiser acquired with breath hold with chemical shift imaging (CSI) matrix overlaid. (B–I) Spectra from the corresponding voxels. Both fit (in red) and raw data (in black) are shown.
**Figure S4.** ^31^P‐MRSI results for a volunteer with a body mass index (BMI) of 20 kg m^−2^. (A) Mid‐short axis GRE localiser acquired with breath hold with chemical shift imaging (CSI) matrix overlaid. (B–I) Spectra from the corresponding voxels. Both fit (in red) and raw data (in black) are shown.
**Figure S5.** ^31^P‐MRSI results for a volunteer with a body mass index (BMI) of 30 kg m^−2^. (A) Mid‐short axis GRE localiser acquired with breath hold with chemical shift imaging (CSI) matrix overlaid. (B–I) Spectra from the corresponding voxels. Both fit (in red) and raw data (in black) are shown.
**Figure S6.** Matlab code and output from the stepwise linear modeling of circumferential strain (CS) using the Akaike Information Criterion (AIC).
**Figure S7.** Matlab code and output from the stepwise linear modeling of radial strain (RS) using the Akaike Information Criterion (AIC).
**Table S1.** Details of the scan protocol and sequences used for our in vivo experiments.
