# Supplementary material for: Regionally resolved cardiac metabolism using a dipole‐loop array coil for 7 T 31P‐MRSI
Source: Magn Reson Med. 2025 Mar 23;94(2):480–96. doi: 10.1002/mrm.30492 (PMC12137756; doi:10.1002/mrm.30492)
Supplement: Supplementary file 1 — Figure S1. Diagram illustrating the different scans that volunteers have undertaken. Seventeen volunteers were scanned in total. Eight volunteers were scanned for the main study only and 9 were scanned for the main study and the reproducibility study. Figure S2. 31P‐MRSI results for a volunteer with a body mass index (BMI) of 34 kg m−2. (A) Mid‐short axis GRE localiser acquired with breath hold with chemical shift imaging (CSI) matrix overlaid. (B–I) Spectra from the corresponding voxels. Both fit (in red) and raw data (in black) are shown. Figure S3. 31P‐MRSI results for a volunteer with a body mass index (BMI) of 26 kg m−2. (A) Mid‐short axis GRE localiser acquired with breath hold with chemical shift imaging (CSI) matrix overlaid. (B–I) Spectra from the corresponding voxels. Both fit (in red) and raw data (in black) are shown. Figure S4. 31P‐MRSI results for a volunteer with a body mass index (BMI) of 20 kg m−2. (A) Mid‐short axis GRE localiser acquired with breath hold with chemical shift imaging (CSI) matrix overlaid. (B–I) Spectra from the corresponding voxels. Both fit (in red) and raw data (in black) are shown. Figure S5. 31P‐MRSI results for a volunteer with a body mass index (BMI) of 30 kg m−2. (A) Mid‐short axis GRE localiser acquired with breath hold with chemical shift imaging (CSI) matrix overlaid. (B–I) Spectra from the corresponding voxels. Both fit (in red) and raw data (in black) are shown. Figure S6. Matlab code and output from the stepwise linear modeling of circumferential strain (CS) using the Akaike Information Criterion (AIC). Figure S7. Matlab code and output from the stepwise linear modeling of radial strain (RS) using the Akaike Information Criterion (AIC). Table S1. Details of the scan protocol and sequences used for our in vivo experiments. [file MRM-94-480-s001.docx]

# Supplementary information

| **Sequence** | **Acquisition time** | **Nucleus** | **Function** |
| --- | --- | --- | --- |
| Localizers | 2 min | ^1^H | Anatomic localization |
| 2,4 chambers and short axis localizers | 40 s | ^1^H | Anatomic localization w/ breath hold |
| Calibration sequences | 20 min | **^31^P** fiducials | Coil localization and transmit efficiency |
| UTE-CSI-FID | 28 min | ^31^P | Heart energetics |
| **Total** | 52 – 60 min | / | / |

Table S1: Details of the scan protocol and sequences used for our in vivo experiments.


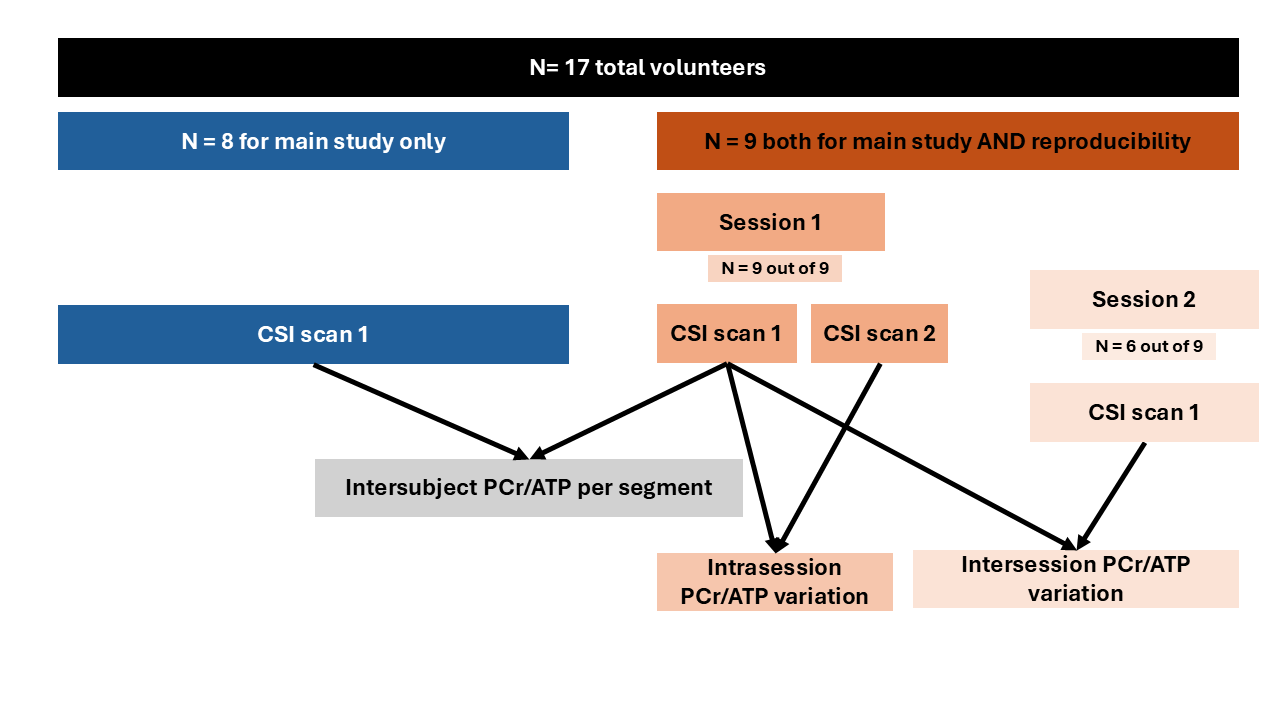


Figure S1: Diagram illustrating the different scans that volunteers have undertaken. 17 volunteers were scanned in total. 8 volunteers were scanned for the main study only and 9 were scanned for the main study and the reproducibility study.


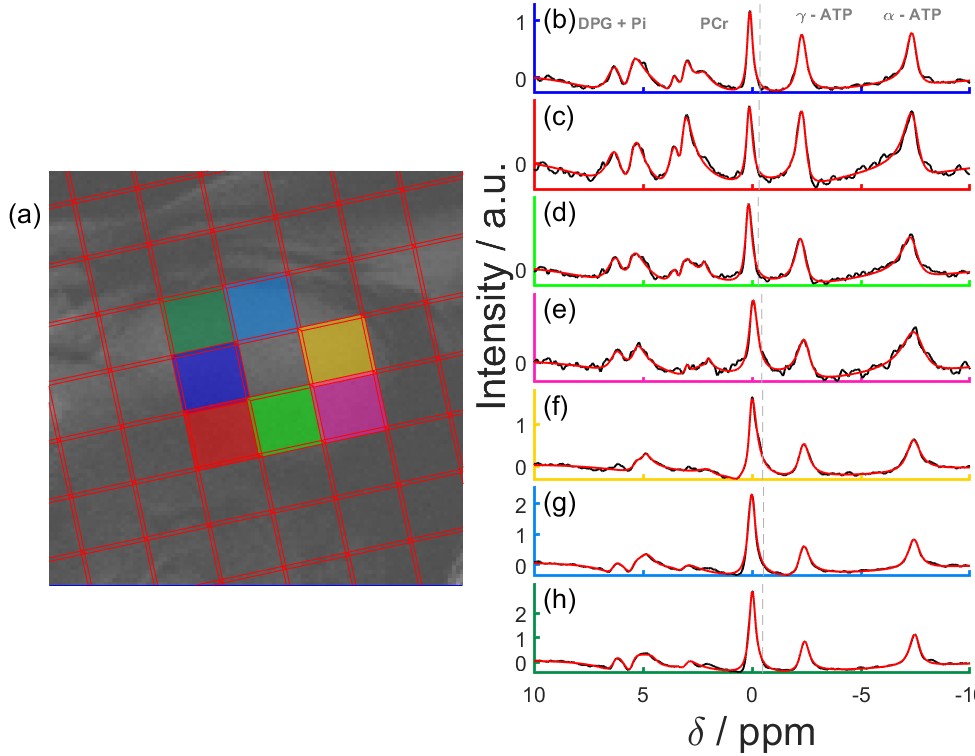


Figure S2: ^31^P-MRSI results for a volunteer with a BMI of 34 kg m^-2^. a) Mid-short axis GRE localiser acquired with breath hold with CSI matrix overlaid. b)-i) Spectra from the corresponding voxels. Both fit (in red) and raw data (in black) are shown.


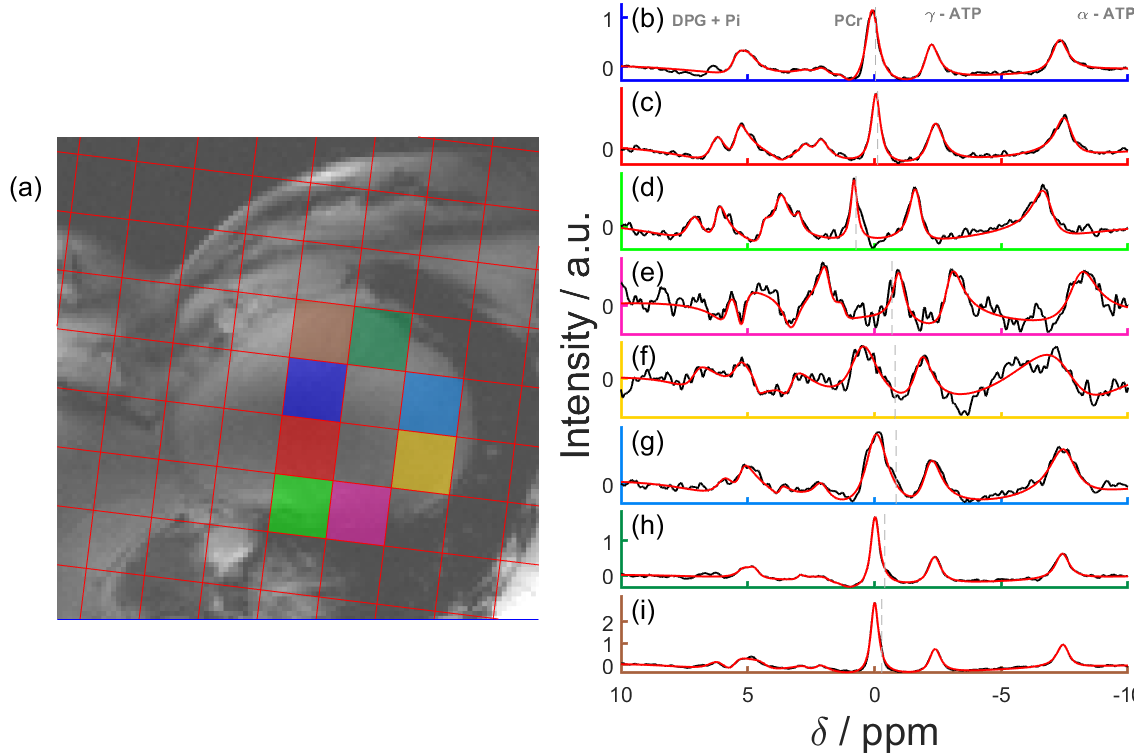


Figure S3: ^31^P-MRSI results for a volunteer with a BMI of 26 kg m^-2^. a) Mid-short axis GRE localiser acquired with breath hold with CSI matrix overlaid. b)-i) Spectra from the corresponding voxels. Both fit (in red) and raw data (in black) are shown.


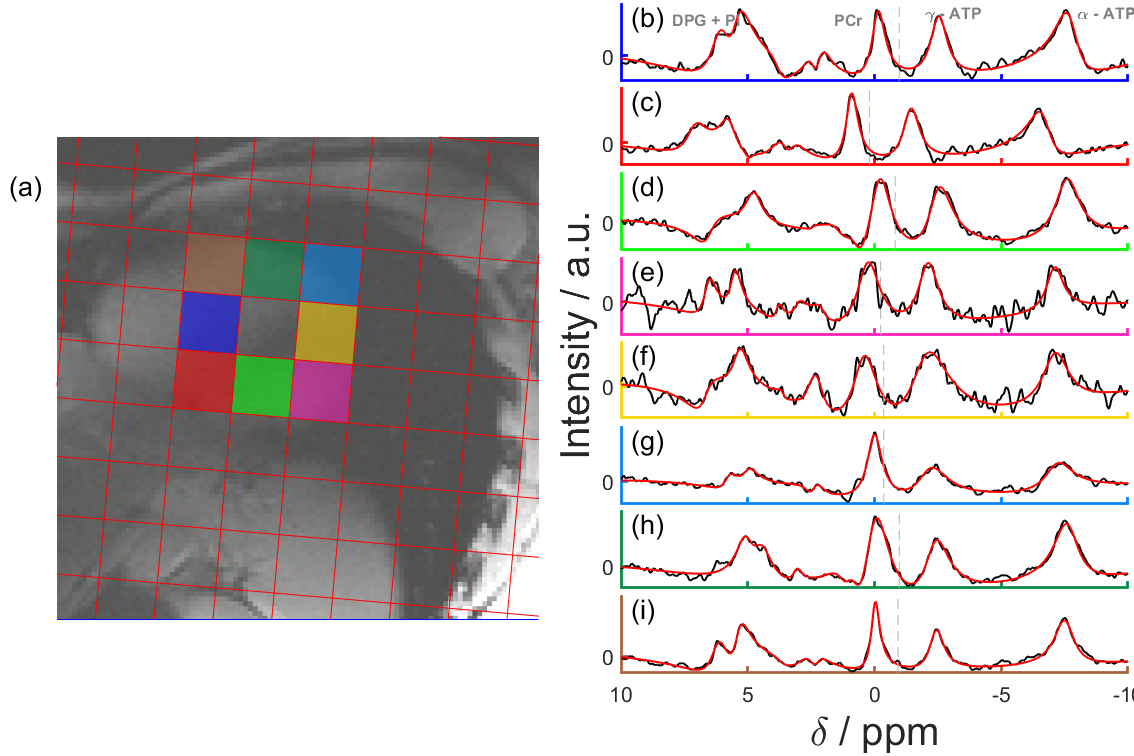


Figure S4: ^31^P-MRSI results for a volunteer with a BMI of 20 kg m^-2^. a) Mid-short axis GRE localiser acquired with breath hold with CSI matrix overlaid. b)-i) Spectra from the corresponding voxels. Both fit (in red) and raw data (in black) are shown.


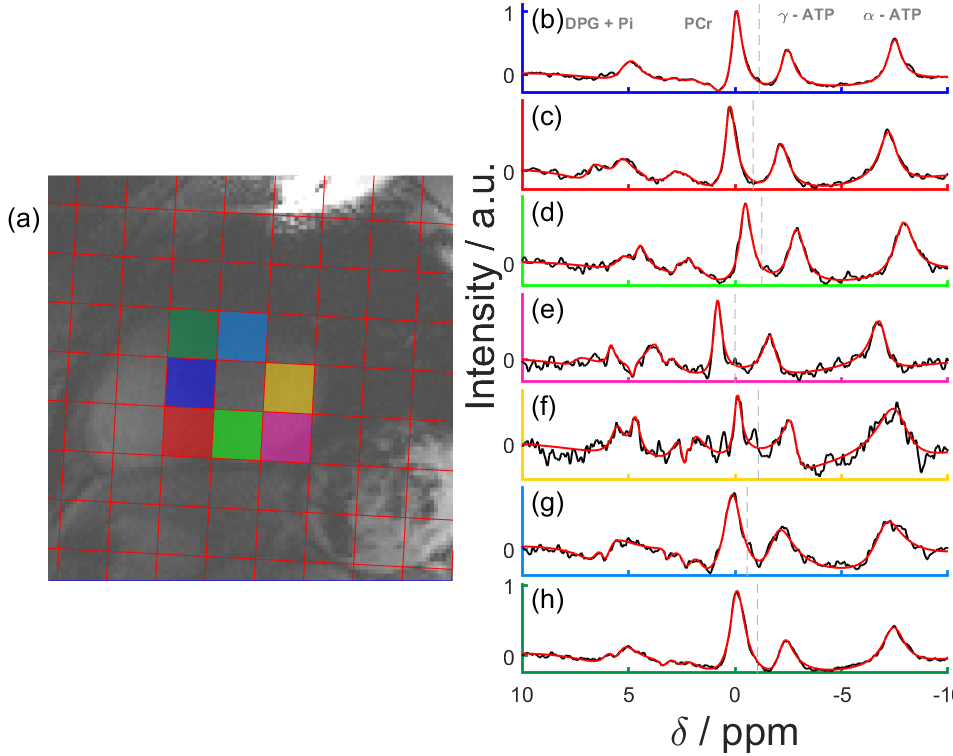


Figure S5: ^31^P-MRSI results for a volunteer with a BMI of 30 kg m^-2^. a) Mid-short axis GRE localiser acquired with breath hold with CSI matrix overlaid. b)-i) Spectra from the corresponding voxels. Both fit (in red) and raw data (in black) are shown.


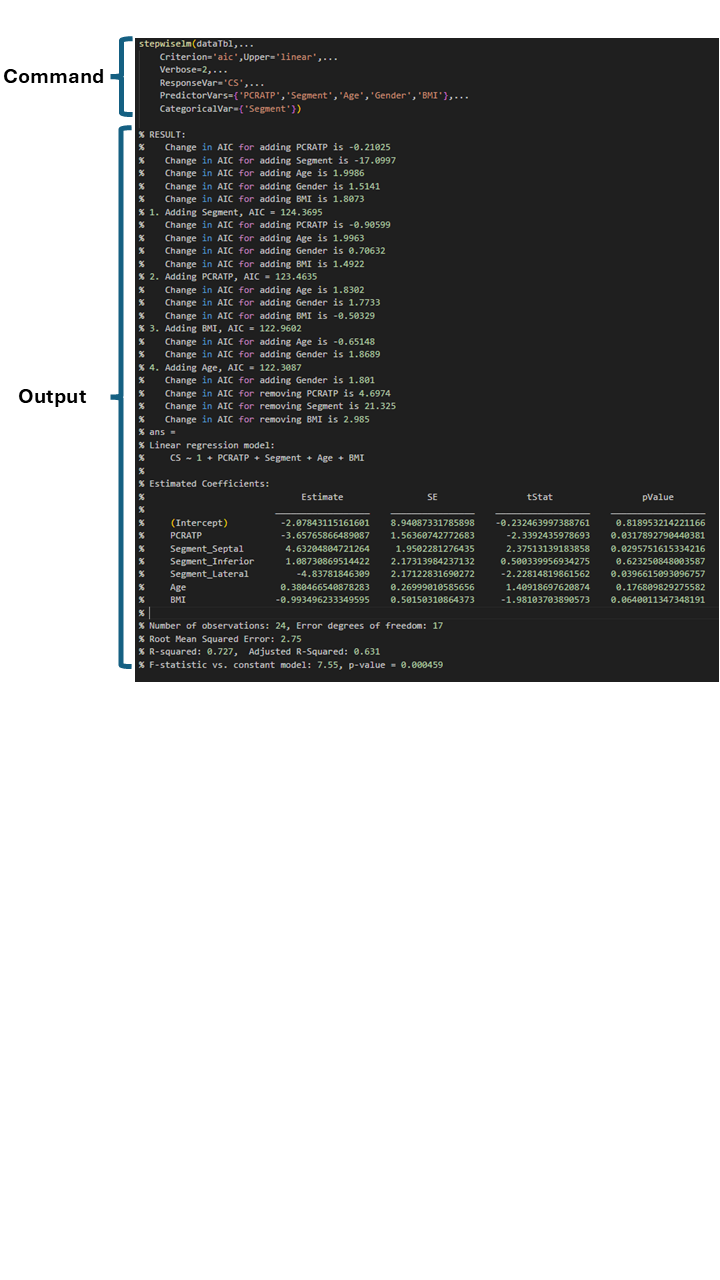


Figure S6: Matlab code and output from the stepwise linear modelling of circumferential strain (CS) using the Akaike Information Criterion (AIC).


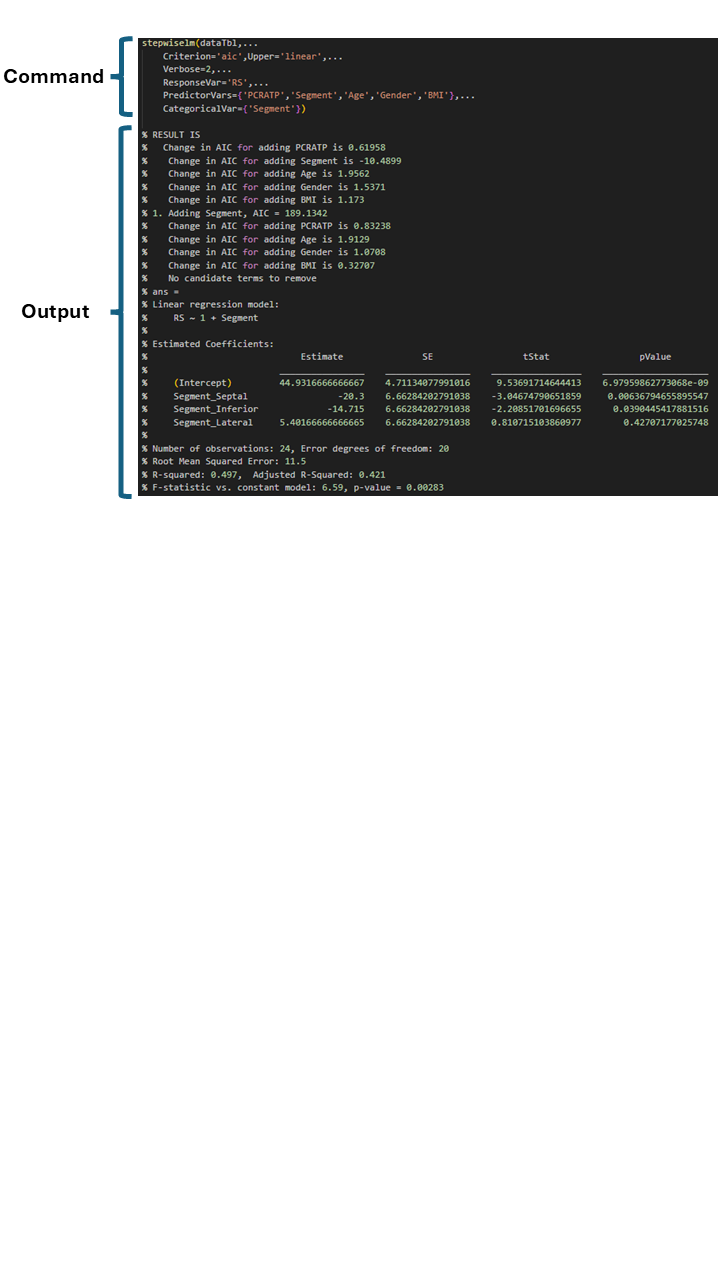


Figure S7: Matlab code and output from the stepwise linear modelling of radial strain (RS) using the Akaike Information Criterion (AIC).
